# Policy Recommendations for Chagas Disease: An Emerging Concern of Public Health Significance for the U.S. Military

**DOI:** 10.4269/ajtmh.26-0052

**Published:** 2026-09-01

**Authors:** Gerardo J. Pacheco, Jose A. Betancourt, Diane Dolezel, Bradley M. Beauvais, Joseph E. Marcus, Paul Lenhart, Paula S. Granados

**Affiliations:** ^1^ School of Health Administration, Texas State University, San Marcos, Texas, USA;; ^2^ Department of Health Informatics and Information Management, Texas State University, San Marcos, Texas, USA;; ^3^ Brooke Army Medical Center, Fort Sam Houston, Texas, USA;; ^4^ Entomological Sciences Division, Public Health Command, West, Fort Sam Houston, Texas, USA;; ^5^ Entomology Branch, Walter Reed Army Institute of Research, Silver Spring, Maryland, USA;; ^6^ School of Public Health, San Diego State University, San Diego, California, USA

## Abstract

Chagas disease, caused by *Trypanosoma cruzi*, poses an emerging and under recognized threat to U.S. military personnel deployed in endemic regions. Despite significant morbidity and mortality risks, the US Department of Defense (DoD) lacks comprehensive policies for Chagas disease prevention, screening, or treatment, unlike other similar infectious diseases. We conducted a comparative policy analysis of existing DoD policies on tuberculosis, HIV, and hepatitis C to inform the development of Chagas disease policy. Using an adapted Political, Administrative, Social, Technological, Economic, Legal factors (PASTEL) framework, we evaluated five policy alternatives through expert consensus scoring. Military health policies and literature were systematically reviewed to identify screening protocols, risk assessment tools, and prevention strategies. Analysis of current military screening policies revealed significant gaps in Chagas disease coverage in comparison with analogous infectious diseases. The decision matrix analysis identified an integrated Periodic Health Assessment (PHA) approach as the optimal policy alternative (weighted score 4.20/5.0), followed by targeted risk-based screening (3.85/5.0). Universal screening scored lowest (2.20/5.0) due to poor cost-effectiveness and implementation challenges. The recommended approach leverages existing annual health assessment infrastructure while incorporating Chagas disease-specific risk evaluation tools. A tiered policy strategy integrating Chagas disease risk assessment into routine military health evaluations offers the most feasible approach for protecting service members. Implementation should prioritize risk-based screening, vector-control measures (e.g., insecticide-treated uniforms), and enhanced surveillance in endemic deployment areas. This framework provides an evidence-based foundation for developing comprehensive DoD Chagas disease policies.

## INTRODUCTION

### Background and domestic burden.

Chagas disease, also known as American trypanosomiasis, is a parasitic infection caused by *Trypanosoma cruzi* that represents a significant but underrecognized public health concern.^1^ Historically considered endemic only in Latin America, the Centers for Disease Control and Prevention (CDC) now recognizes the disease as present within the United States, rather than formally endemic.^2^ An estimated 300,000 individuals in the United States are living with chronic infection.^1^^,^^3^^–^^5^ Recent modeling clarifies that approximately 288,000 of these infections occur among Latin American–born adults, whereas an estimated 10,000 represent locally acquired (autochthonous) cases, reflecting ongoing, albeit limited, domestic transmission.^2^ The full U.S. burden remains unknown because systematic surveillance is limited to only eight states.^2^

### Transmission, clinical progression, and health care gaps.

Transmission occurs primarily via triatomine insects, whose feces can enter the body through bites, mucosa, or conjunctiva.^4^ Other routes of infection include congenital, transfusion, organ transplant, foodborne exposure, and laboratory accidents.^6^^,^^7^ The infection progresses from a brief, largely asymptomatic acute phase to a chronic phase that is lifelong if left untreated.^5^^,^^8^ If undetected and untreated, 20% to 30% of chronically infected individuals develop severe cardiac or gastrointestinal disease, including cardiomyopathy, heart failure, or sudden death.^5^ In the United States, delayed diagnosis is driven not only by limited surveillance infrastructure but also by low clinical awareness among health care providers,^9^ many of whom do not routinely screen high-risk populations or recognize the potential for domestic vector-borne transmission.^10^

### Domestic exposure risk at U.S. military installations.

U.S. military personnel are uniquely vulnerable to *T. cruzi* exposure in the environments in which they train and deploy.^11^^,^^12^ These environments frequently include rural, austere, or substandard conditions that facilitate contact with triatomine vectors. The parasite thrives in underdeveloped environments and substandard structures, settings where military forces frequently operate. The severe consequences of these environmental exposures were highlighted by a recent outbreak of Chagas disease among 11 Colombian soldiers after foodborne exposure to *T. cruzi* via contaminated açaí palm juice (“chucula”) into which triatomine insects or their feces had been introduced during processing; two soldiers died, and seven required hospitalization for serious cardiac complications, including pericardial effusion.^13^

U.S. military personnel remain at risk whether deployed domestically or abroad. Triatomine vectors occur in at least 30 U.S. states, particularly across the South where many large military installations are located; published maps of triatomine distribution and autochthonous *T. cruzi* transmission risk in the continental United States provide a useful geographic reference for identifying installations of concern.^14^ Highlighting the domestic risk within military contexts, Webber et al.^12^ documented *T. cruzi* infections among military working dogs at a major training base in Texas, raising significant public health concerns for both animals and personnel sharing the same operational environment. Furthermore, a case study in a *T. cruzi*–positive young U.S. military veteran demonstrates the risk of repeated triatomine exposures during service.^11^ Because there is currently no cure for advanced disease and no available vaccine, early screening and vector mitigation are critical to protecting military force readiness. These findings collectively suggest that Chagas disease represents not only an imported infectious risk but also a plausible occupational exposure within domestic military training environments.

### Deployment-related exposure in endemic regions.

The US Department of Defense (DoD) moves personnel globally through temporary duty, permanent change of station, and deployment orders. Service members (SMs) may be exposed to *T. cruzi* in endemic regions during a short-term assignment or deployment tour (several days to 9 months) or longer permanent change of station assignments (3–5 years or more). Notable examples include the US Navy Medical Research Unit South (NAMRU-South), which has historically operated across South America, and ongoing Special Operations rotations throughout Central and South America, regions with well-documented sylvatic *T. cruzi* transmission cycles. Operational deployments to endemic regions introduce sustained exposure risk, particularly in environments where vector control is limited and field conditions mirror those associated with known transmission cycles.

### Military recruits from endemic regions.

Military accession (entry into service) provides a unique public health opportunity to identify previously undiagnosed Chagas disease among recruits. Many individuals with chronic infection remain asymptomatic for decades and may be unaware of their condition at the time of entry into service.

The Military Health System (MHS) offers a structured, centralized platform for screening, diagnosis, and treatment that may not otherwise be available in civilian settings. Accordingly, this population should be framed not solely as a risk group (if originating from endemic regions or the southern United States) but also as an opportunity for early detection and intervention. However, screening policies must be implemented carefully to ensure that they facilitate access to care rather than inadvertently triggering administrative separation during early accession periods.

### Military health policies.

The DoD currently maintains several infectious disease^15^^–^^19^ policies that provide guidance on treatment protocols and the medical disposition of SMs diagnosed with these conditions. Medical disposition refers to the formal determination of a service member’s fitness for continued service, including evaluation of duty limitations, deployability, and long-term medical requirements.^16^

The DoD annually updates its list of infectious diseases of operational importance—medical threats that can impair the effectiveness of deployed forces in both the short and long term. Despite its clinical severity and potential impact on force readiness, Chagas disease is not currently included on this list and lacks formal policy guidance within the MHS.

Military health policies must balance multiple competing priorities: maintaining force readiness, protecting individual service member health, ensuring global deployability, and managing health care costs. The U.S. military comprises six service branches: the Army, Navy, Marine Corps, Air Force, Space Force, and Coast Guard. Each operates under a distinct doctrine, deployment profiles, and health care delivery structures. The MHS provides care through both direct care facilities (military treatment facilities) and the TRICARE managed care program. For the purposes of this analysis, Army, Navy/Marine Corps, and Air Force policies are examined, because these branches have the largest deployed footprints in Chagas disease–endemic regions and maintain the most detailed infectious disease screening documentation. Existing infectious disease policies (e.g., tuberculosis [TB], HIV, and hepatitis C [HCV]) illustrate how the DoD operationalizes these priorities through risk-stratified screening, surveillance, and management strategies tailored to disease-specific characteristics and transmission dynamics.

These established policy frameworks provide a practical foundation for evaluating how Chagas disease screening and prevention strategies could be integrated into existing military health systems without disrupting operational workflows. Understanding these established policy frameworks provides essential insights for developing effective Chagas disease management strategies that align with military operational needs and health care delivery capabilities.

### Public health relevance of Chagas disease for the U.S. military.

Chagas disease is an emerging public health concern in the United States.^20^ As of 2025, Chagas disease is reportable in eight states, including Arizona, Arkansas, Louisiana, Mississippi, Utah, Tennessee, Texas, and Washington, which limits the ability to comprehensively monitor domestic trends and detect local transmission across the broader United States.^21^^,^^22^ This fragmented surveillance infrastructure is further compounded by limited awareness among health care providers, many of whom are unfamiliar with appropriate screening practices, diagnostic pathways, and clinical management of Chagas disease.^23^ Because the infection often remains asymptomatic for decades, individuals rarely seek screening, allowing the disease to progress undetected until serious cardiac or gastrointestinal complications arise.

In the military context, these surveillance and awareness gaps create a heightened risk that service members may acquire infection without detection, with clinical consequences emerging later in their careers and potentially impacting long-term readiness and health care utilization. The DoD currently lacks comprehensive health policies for Chagas disease. To inform policy development, we conducted a comparative analysis of Chagas disease alongside TB, HIV, and HCV, which share key characteristics including chronic disease progression, asymptomatic phases, and operational relevance to military populations. Like Chagas disease, these diseases require long-term management and jeopardize readiness if undiagnosed.

## MATERIALS AND METHODS

### Literature review for screening and testing policies.

The study team examined the body of related literature to identify existing DoD policies specific to Chagas disease. The search was conducted by three researchers using public databases (e.g., PubMed, Google Scholar) and military resources (e.g., military and academic peer-reviewed journals, DoD doctrine databases) to identify military protocols, military-focused policies, and military guidance for the three proxy diseases. TB, HIV, and HCV were selected as comparator conditions based on shared characteristics with Chagas disease, including chronic disease progression, asymptomatic phases, and relevance to military screening and surveillance practices. The goal was to extract practical, feasible, and evidence-based strategies to inform Chagas disease policy development in the U.S. military context. To ensure consistency in evaluation, each disease was examined across three predefined domains: 1) screening policies and testing requirements, 2) risk assessment protocols, and 3) prevention and surveillance strategies.

### Screening and testing framework.

Military screening protocols serve dual purposes: maintaining force readiness and preventing disease transmission within military populations. Screening identifies asymptomatic individuals who may harbor infectious diseases, whereas testing confirms suspected cases through diagnostic evaluation.^24^ Military personnel undergo medical evaluations at accession and periodically throughout their careers to identify conditions that could impair duty performance or pose transmission risks. These evaluations help maintain medical fitness, ensure deployment readiness, enable early disease detection, and support compliance with military health standards.

The DoD categorizes medical findings based on timing of discovery. Conditions identified within the first 180 days of service fall under accession standards, where individuals not meeting medical criteria (such as newly diagnosed TB or HCV) are often administratively separated. Diagnoses made after 180 days fall under retention standards, where service members may continue serving if asymptomatic and deemed deployable, but face separation if their condition prevents worldwide duty assignment. This distinction between accession and retention standards is particularly relevant for Chagas disease policy development, because the timing of diagnosis may have significant implications for both access to care and service member retention. Although the DoD annually updates its list of infectious diseases of operational importance, Chagas disease is not included and lacks formal policy guidance.

### Policy analysis of disease.

Table 1 summarizes screening and testing policies across service branches for the three comparator diseases. The analysis reveals significant variation in approaches, with targeted testing predominating for TB, universal screening required for HIV, and limited routine screening for HCV. These variations reflect differing assessments of disease risk, transmission dynamics, and operational impact across conditions, providing a comparative framework for evaluating the feasibility and appropriateness of potential Chagas disease screening strategies within the MHS.

**Table 1:** Summary of service branch policies for analogous diseases

Disease	Branch	Accession Testing	Periodic Testing	Deployment Testing	Post-Deployment Testing
TB	Army	Targeted	Risk-based*	No	No (PHA only)
	Air Force	Targeted	Risk-based^†^	No	No
	Navy	Universal for LTBI	Annual risk screening^‡^	Only if exposed or deemed high risk	Only if exposed or deemed high risk
HIV	All	Universal	Every 2 years and predeployment	Yes	N/A
HCV	Army	Not required	Not routine	Not routine	Not routine
	Navy	Required	Not routine	Not routine	Not routine
	Air Force	Not required	Not routine	Not routine	Not routine

HCV = hepatitis C virus; LTBI = latent TB infection; PHA = periodic health assessment; TB = tuberculosis.

* Certain career fields require additional testing in close-quarters environments.

^†^ No frequency specified in policy.^25^

^‡^ Annual questionnaire screening with testing only if high-risk factors identified.

#### TB screening.

Between 1998 and 2012, the U.S. military reported 128 TB cases,^26^ reflecting low prevalence and incidence among SMs.^27^ Consequently, TB screening policies emphasize targeted approaches for high-risk populations.^16^ The Army recommends risk-based testing during accession and annual assessments of risk, discouraging universal testing to minimize false positives.^16^ Air Force protocols limit testing to SMs with high-risk exposures or high-risk occupations (e.g., health care workers), or clinical indications.^19^ The Navy requires universal latent TB infection (LTBI) screening for all personnel unless previous TB infection is documented, with annual risk assessment questionnaires determining the need for follow-up testing.^28^ This variation across service branches reflects differing operational risk environments and illustrates a flexible, risk-based screening model that prioritizes efficiency while maintaining force health protection.

### HIV/AIDS screening.

Despite relatively low military HIV incidence (23 per 100,000 in 2021, in comparison with approximately 13 per 100,000 in the U.S. general population),^17^ the DoD requires screening for all military applicants prior to accession for all of the Service branches, with mandatory testing every 2 years.^15^^,^^17^ Until a recent court decision,^29^ individuals with an HIV-positive status were excluded from service during accession.^30^ Currently, SMs are not disqualified solely based on HIV status.^17^ This universal screening approach reflects the DoD’s prioritization of diseases with long-term management implications and potential readiness impacts, supported by established treatment pathways and surveillance systems. Service branch variations in HIV screening intensity may also reflect differences in operational environments (e.g., sustained close-quarters conditions aboard naval vessels versus shorter-duration field deployments) as well as the lasting institutional response to the HIV epidemic of the 1980s and 1990s, which prompted robust and enduring DoD-wide screening infrastructure.

### HCV virus screening.

Military HCV screening policies show the greatest inconsistency across services. Although the CDC recommends universal adult HCV screening at least once and subsequent screening for pregnant women during each pregnancy, periodic screening is recommended for high-risk individuals.^31^ Surveillance data demonstrate a reduction in new HCV cases among SMs between 2011 and 2020 (4.8 to 1.6 per 100,000 person/years).^32^^,^^33^ However, higher incidence rates have been noted in Navy military members, health care workers (in comparison with combat military members), and those with one prior deployment (in comparison with no deployments or multiple deployments).^33^ Before entry into military service, accession applicants must disclose their medical history, including prior diagnoses of HIV, TB, and HCV infection.^32^ Applicants are considered medically ineligible if they have a current acute or chronic hepatitis carrier state, have experienced clinically apparent hepatitis within the preceding 6 months, or show evidence of liver function impairment.^33^ Routine HCV screening within the military is limited. Only the Navy and Marine Corps^18^ require routine serological testing.^18^ The Air Force screens for hepatitis A and B immunity during Basic Military Training (BMT) but does not screen for hepatitis A, hepatitis B, or HCV infection,^34^ and the Army does not require initial or routine HCV screening.^33^ This inconsistency highlights the absence of a standardized DoD-wide approach for certain chronic infections, underscoring how variability in perceived risk and operational priorities can lead to fragmented screening policies.

### Risk assessment and surveillance framework.

Public health surveillance, defined as the systematic and continuous collection, analysis, and interpretation of health-related data, is essential in monitoring and preventing disease.^35^ The disease burden among specific populations and the related risk factors are monitored and tracked through public health surveillance efforts. Although passive surveillance systems rely on automated disease reporting, active systems requiring more rigorous infrastructure may be used to collect data on rare diseases.^35^ The cornerstone of military public health surveillance is force health protection.^36^ Risk assessment is the ongoing and methodical process to determine whether individuals are at risk for disease.^16^ The scope of relegated public health services varies by service branch. The branch-specific risk assessment tools and protocols for each disease are summarized in Table 2.

**Table 2:** Periodic risk assessment tools and protocols

Disease	Branch	Risk Assessment Tool Used	Application
TB	Army	MEDCOM Form 830	PHA, separation
	Navy	NAVMED 6,224/8	Annual PHA
	Air Force	Aerospace Medicine Council guidance	High-risk only
HIV	All Branches	None formalized	Indirect risk monitoring
HCV	All Branches	CDC Guidelines and VA reflex testing	Applied situationally (e.g., combat injuries)

CDC = US Centers for Disease Control and Prevention; HCV = hepatitis C virus; PHA = periodic health assessment; TB = tuberculosis.

Although TB, HIV, and HCV are all reportable conditions supported by established surveillance mechanisms, the intensity of active surveillance and prevention efforts varies substantially across diseases, reflecting differences in perceived threat and operational impact. The levels of active surveillance and prevention measures are summarized in Table 3.

**Table 3:** Prevention and surveillance strategies

Disease	Personal Protective Measures	Education	Active Surveillance	Passive Surveillance
TB	N95 respirators for health care workers in high-risk settings	Limited	Contact investigations	Reportable disease
HIV	Condom promotion, needle safety; PrEP	Moderate to Strong	Contact investigations; behavioral monitoring; HRBS analyses	Reportable disease
HCV	Needle safety, universal precautions	Limited to Moderate	No active DoD-wide	Reportable disease

HCV = hepatitis C virus; PrEP = preexposure prophylaxis; HRBS = health-related behaviors survey; TB = tuberculosis.

### TB.

The Army’s comprehensive TB surveillance and control program specifies the use of Form 830 as a risk assessment tool (RAT) for all personnel during annual periodic health assessments, with those requiring further testing if risk factors are present (e.g., targeted testing) if the SM responds “yes” to any of the risk factors.^16^ Separation, defined as the release from active duty, discharge, retirement, dismissal, or resignation, occurs as the result of SM noncompliance to treatment, active or persistent TB infection that affects SM’s fitness for continued service, or cases in which LTBI complications arise.^16^ Similarly, NAVMED 6224/8 is used to assess the risk of exposure to TB among Navy personnel.^28^ However, the Air Force lacks a RAT for TB. Limited education on TB is available to SMs as they complete the PHA RAT. TB is a reportable medical event (RME)^37^ included in the MHS’s Disease Reporting System internet (DRSi). Early detection remains a salient approach across the service branches in preventing TB.

### HIV.

According to DoD-wide policy, all SMs undergo HIV screening every 2 years during periodic health assessments.^17^ As described by Aumakhan et al.,^38^ initatives to curtail new cases of HIV^17^ in the military remains a challenge, despite the wide use of preexposure prophylaxsis (PrEP) in high-risk individuals and immediate treatment after confirmatory testing. The DoD maintains the need to raise SM awareness and education and continue screening initiatives as strategic efforts to reduce HIV transmissions by 90% by 2030.^17^ Nonetheless, each service follows specific doctrine. For example, the Navy’s Instruction 5300.30F^18^ outlines the public health surveillance and education activities for HIV and HCV. No standardarized risk assessment tool for HIV has been adopted across all service branches. HIV data are monitored in the Defense Medical Surveillance System but are not reported to the DRSi. Additionally, risk factors are monitored through the DoD Health Related Behaviors Survey (HRBS). HIV education efforts focus on awareness and reduction of high-risk behaviors.

### HCV.

No standardized risk assessment tools are specified for HCV. Instead, service branches rely on CDC guidance and situational screening practices. HCV cases^33^ are tracked as RME^37^ in the DRSi. The absence of formalized RATs for HCV further illustrates variability in surveillance intensity across diseases and highlights potential gaps in systematic identification of asymptomatic infections. Recent findings^39^ suggest considering expanding current screening to reduce the number of missed diagnoses, especially during accession.

### Policy decision matrix framework.

To compare alternative DoD policies for Chagas disease prevention and screening, we constructed a structured decision matrix grounded in an adapted Political, Administrative, Social, Technological, Economic, and Legal factors (PASTEL)^40^^,^^41^ lens. PASTEL is widely used in military‐health planning because it captures the full range of external and internal constraints on implementation (e.g., congressional direction, service-level budgets, force health protection mandates). After piloting other multicriteria tools (SWOT [Strengths, Weaknesses, Opportunities, Threats] analysis; simple cost‐utility ranking), the team selected PASTEL because it: 1) aligns with the DoD’s Joint Health Services Doctrine, 2) mirrors evaluation formats already familiar to line-medical commanders, and 3) supports transparent weighting of heterogeneous criteria.^42^

Five policy alternatives, Status Quo, Targeted Risk Based Screening, Universal Screening, Integrated Periodic Health Assessment (PHA), and the Comprehensive Prevention Program, were arrayed against the six PASTEL dimensions (Table 4). The five alternatives are defined as follows: 1) Status Quo is the continuation of current practice with no formal Chagas disease screening or policy; 2) Targeted Risk-Based Screening is serological testing offered to service members with identifiable risk factors, such as birth or extended residence in endemic regions or documented vector exposure; 3) Universal Screening is mandatory serological testing for all accession applicants and active-duty personnel regardless of risk history; 4) Integrated PHA is incorporation of a standardized Chagas disease risk assessment questionnaire into existing annual PHA workflows, with confirmatory testing triggered by positive risk factors; and 5) the Comprehensive Prevention Program is a broad initiative combining screening, vector control, education, and policy reform across all service branches. For each criterion, alternatives were scored independently by the investigators using a 5-point ordinal scale (1 = very poor, 3 = acceptable, and 5 = excellent). Prior to scoring, the panel calibrated the scale by benchmarking against existing DoD policies for TB, HIV, and HCV; for example, a score of 5 in “Administrative Feasibility” corresponds to an intervention that could be executed with the same resource footprint as the current HIV testing program, whereas a score of 1 would require new billets and laboratory expansion (Table 4).

**Table 4:** Definitions of ranked PASTEL criteria

Criterion	1 = Very Poor	3 = Acceptable	5 = Excellent
Political feasibility	Requires new legislation and unfunded mandate	Needs a secretary-level policy memo	Fits entirely within existing directives and budgets
Administrative feasibility	New billets/laboratory expansion required	Re-tasks existing staff with moderate workflow change	Drops into current PHA or clinic workflows with no extra manpower
Social acceptability	High stigma or personal burden; likely refusals	Neutral troop perception	Clear personal benefit; negligible stigma or burden
Technological requirements	Relies on unfielded or experimental tech	Minor laboratory upgrades or new supply items	Uses FDA-cleared assays already stocked by the Defense Logistics Agency
Economic impact	Large per year net cost, minimal offset	Cost-neutral over 5 years	Cost-saving within 5 years
Legal compliance	Conflicts with federal statute or SOFA	Requires Office of General Counsel review	Fully compliant with existing law and regulation

FDA = US Food and Drug Administration; PASTEL = Political, Administrative, Social, Technological, Economic, Legal factors; PHA = periodic health assessment; SOFA = status of forces agreements.

Criterion weights (Table 5) were established through consultations with the advisory team, two retired military preventive medicine officers, one active-duty infectious-disease physician, and two civilian Chagas experts. The group agreed on the final weights by consensus. The same team assigned scores of 1 to 5 for each policy-criterion cell; if two scores differed by more than 1 point, the midpoint was used. A quick sensitivity check with equal weights produced the same top two policy choices (Integrated PHA and Targeted Screening).

**Table 5:** Evaluation criteria and weights (adapted PASTEL framework)

Criterion (PASTEL element)	Weight*	Operational Definition in the DoD Context	DoD Relevance
Political feasibility	0.25	Probability that senior DoD leadership and Congress will authorize and resource the policy	Readiness-enhancing policies receive the strongest backing and are rarely blocked once endorsed
Administrative feasibility	0.20	Fit with Military Health System personnel, clinics, laboratories, and data systems	Clinic throughput and staffing ceilings are chronic constraints; overloading them erodes readiness
Social acceptability	0.15	Likelihood that service members and beneficiaries will cooperate with the program	Uptake of voluntary testing hinges on troop acceptance; stigma can harm participation
Technological requirements	0.15	Availability and supply-chain robustness of diagnostics, vector-control tools, and EHR modules	DoD logisticians must be able to field approved assays worldwide without delaying deployments
Economic impact	0.15	Net present value to DoD (program cost minus medical-readiness savings)	Budget competition is intense but not overriding when readiness gains are demonstrable
Legal compliance	0.10	Consistency with U.S. law, international agreements, and existing DoD/VA regulations	True legal barriers are uncommon but, when present, are absolute vetoes

DoD = US. Department of Defense; EHR = electronic health record; PASTEL = Political, Administrative, Social, Technological, Economic, Legal factors.

* Weights sum to 1.00.

### Scoring procedure.

Each investigator assigned a rating for every (alternative × criterion) cell guided by the anchors above and documented qualitative evidence (e.g., diagnostic throughput studies, cost models, etc.). The weighted totals determined rank order; ties were broken by selecting the option with the higher unweighted mean score.

### Basis for weight applicability for the DoD population.

The final scheme mirrors institutional priorities outlined in the 2024 MHS Strategic Plan, where 1) political support/readiness, 2) administrative executability, and 3) cost-effectiveness are the dominant decision drivers.^43^ Sensitivity tests confirmed that ± 10% shifts in any single weight did not change the preferred option, supporting robustness.

## RESULTS

### Policy decision matrix analysis.

The policy evaluation matrix analysis identified significant differences among the five policy alternatives for Chagas disease management in the U.S. military (Table 6). The Integrated PHA approach achieved the highest weighted score (4.20), followed by Targeted Risk-Based Screening (3.85). Universal Screening received the lowest score (2.20), driven primarily by unfavorable economic impact and lower administrative feasibility scores relative to alternative strategies.

**Table 6:** Policy decision matrix for Chagas disease screening alternatives

Policy Alternatives						
Evaluation Criteria	Weight	Status Quo	Targeted Screening	Universal Screening	Integrated PHA	Comprehensive Program
Political feasibility	0.25	3	4	2	4	3
Administrative feasibility	0.20	5	4	2	5	3
Social acceptability	0.15	3	4	3	4	4
Technological requirements	0.15	5	3	2	4	3
Economic impact	0.15	4	4	1	4	2
Legal compliance	0.10	2	4	4	4	4
Total weighted score*	1.00	3.75	3.85	2.20	4.20	3.10
Rank	–	3	2	5	1	4

PHA = Periodic Health Assessment.

* Scoring: 1 = Very Poor, 3 = Acceptable, 5 = Excellent.

### Key findings by criterion.

The rows in Table 6 represent the evaluation criterion, which are discussed in this section.

### Political feasibility.

Both Targeted Screening and Integrated PHA approaches scored highest (4.0), because they build on existing military health policy frameworks for TB and HIV screening. Universal Screening scored lowest (2.0) because of anticipated resistance from budget constraints and implementation complexity. These findings suggest that policy options aligned with existing DoD screening paradigms are more likely to gain institutional support and be implemented without substantial policy or legislative barriers.

### Administrative feasibility.

The Integrated PHA approach and Status Quo scored highest (5.0), with the PHA option leveraging existing annual health assessment infrastructure. Universal Screening scored lowest (2.0) because of significant resource and personnel requirements.

### Economic impact.

Status Quo, Targeted Screening, and Integrated PHA approaches all scored well (4.0), demonstrating cost-effectiveness. Universal Screening scored poorly (1.0) because of high ongoing testing costs, whereas the Comprehensive Program scored moderately (2.0) due to higher up-front investment requirements. These results indicate that strategies incorporating targeted or integrated screening approaches are more economically sustainable within the constraints of the MHS. Undetected and untreated Chagas disease imposes substantial long-term costs, including cardiomyopathy management; disability separation pay; and the loss of experienced, highly trained service members, costs that proactive screening is designed to mitigate.

### Policy recommendation.

Based on the decision matrix analysis, the Integrated PHA approach is the recommended policy alternative for DoD Chagas disease screening and prevention. This approach scored highest across multiple criteria while maintaining strong feasibility for implementation within existing DoD health care systems. This approach consistently performed well across multiple evaluation criteria, demonstrating a balanced profile of political feasibility, administrative feasibility, and economic sustainability while maintaining strong potential for improving early detection of Chagas disease.

### Scoring rationale for policy alternatives.

The scores were not arbitrary; they reflect graded judgments based on how each alternative compares to current practices, known barriers, and analogs from existing DoD disease management strategies. Team members discussed each criterion individually, debated expected implementation challenges and stakeholder responses, and reached consensus on final values. The use of structured criteria and consensus-based scoring enhances the transparency and reproducibility of the evaluation, while grounding the analysis in established DoD policy frameworks and operational realities.

## DISCUSSION

### Policy implementation considerations.

The decision matrix analysis supports implementing a tiered approach to Chagas disease screening that integrates with existing DoD health assessment protocols. The Integrated PHA approach achieved the highest overall score and demonstrated consistent performance across key feasibility domains, reflecting several strategic advantages. Just as TB risk assessment protocols are already integrated across all service branches, a Chagas disease risk assessment questionnaire can be incorporated into annual PHAs without requiring new administrative infrastructure. This approach mirrors the integration of HIV behavioral risk screening into routine military health evaluations.^44^ Unlike universal screening approaches that would require significant laboratory capacity expansion, the integrated approach uses targeted testing based on risk assessment findings. This strategy aligns with current TB screening practices that emphasize risk-based rather than universal testing, as demonstrated in Army and Air Force policies.^16^^,^^26^ An important element is that this approach leverages existing infrastructure to expand surveillance capacity while minimizing disruption to clinical workflows and operational readiness.

The PHA integration ensures systematic risk evaluation for all SMs while maintaining operational readiness. This approach addresses the challenge identified in the Colombian military outbreak,^13^ where lack of awareness contributed to severe outcomes among deployed personnel. The matrix analysis suggests a phased implementation strategy: 1) develop standardized Chagas risk assessment tools, 2) pilot integration in high-risk geographical areas, 3) expand to all service branches with targeted testing protocols, and 4) establish surveillance and reporting mechanisms consistent with existing infectious disease monitoring systems. This phased approach allows for incremental implementation, evaluation, and refinement, reducing risk while facilitating scalable adoption across the MHS. An additional co-benefit of systematic screening within the MHS is its potential to expand the currently limited national epidemiological surveillance data on Chagas disease in the United States, data essential for informing federal public health resource allocation and policy.

An often overlooked but important component of existing DoD infectious disease infrastructure is the military blood donor screening program. Unlike commercial blood donor programs such as the American Red Cross, which screen individual donors only once for *T. cruzi* antibodies upon entry into the donor pool, the DoD requires serological testing at every military blood donation. This more rigorous standard has resulted in the identification of seropositive SM donors who may not have been captured through routine health assessments.^12^^,^^45^ However, this blood donor screening approach does not constitute systematic, population-level surveillance of the SM population; it identifies individuals who happen to donate blood rather than those at highest occupational risk. As such, although military blood donor screening represents an important existing mechanism for case detection, it should be viewed as a complement to, not a substitute for, the comprehensive, risk-based screening framework recommended in this analysis.

The feasibility of integrating Chagas disease screening into an MHS is further supported by experience in other countries where the disease is endemic. The Brazilian Armed Forces have incorporated Chagas disease into their pre-enlistment medical evaluation protocols, reflecting the high seroprevalence among recruits from endemic rural regions, particularly in the northeastern states. Similarly, in Colombia, the 2021 military outbreak^13^ prompted heightened institutional attention to vector-borne disease risk in military training environments. These international examples demonstrate that MHSs can operationalize Chagas disease screening protocols within existing accession and periodic health frameworks and that doing so yields clinically meaningful case detection. The DoD’s well-established infrastructure for managing other chronic infectious diseases, including the global logistics and data systems reviewed in this analysis, positions it to implement analogous protocols with comparable feasibility.

In addition to risks associated with military training and operational activities in endemic regions, accession screening could identify recruits with previously unrecognized *T. cruzi* infection acquired prior to military service through residence in endemic areas or congenital transmission. Such screening would provide an opportunity to diagnose individuals with chronic indeterminate Chagas disease who may otherwise remain undetected for years. An important consideration is that most individuals identified through screening would likely be asymptomatic and without evidence of clinically significant disease. Consequently, infection alone would not be expected to adversely affect military readiness or preclude service. Rather, early identification would allow the MHS to provide appropriate confirmatory testing, clinical evaluation, and antiparasitic treatment when indicated, potentially reducing the risk of future disease progression and supporting force health protection and long-term readiness.

A critical policy consideration for the timing of Chagas disease screening within the military accession process is the 180-day threshold distinguishing accession from retention standards. As described in the “Methods” section, SMs diagnosed with a disqualifying condition within the first 180 days of service may be administratively separated before completing treatment. This determination for separation has occurred previously in the context of other infectious disease diagnoses and carries significant equity implications for Chagas disease screening. Many recruits from endemic regions originate from communities with limited health care access; if screened and identified as seropositive within the first 180 days, they risk administrative separation prior to receiving treatment, leaving them potentially worse off than if they had never been screened.^46^ Accordingly, this analysis recommends that Chagas disease screening protocols be designed to align with retention rather than accession standards wherever operationally feasible, ensuring that identified cases are guaranteed access to treatment through the MHS prior to any separation determination. This approach is consistent with the DoD’s broader commitment to equitable health care for all service members and would maximize the public health benefit of expanded screening.^46^

### Limitations of the policy evaluation matrix approach.

The policy evaluation relied on expert assessment and existing literature, because operational data for Chagas screening programs in military populations are limited. As a result, the analysis reflects informed judgment rather than empirical validation, and findings should be interpreted in that context. The weighting scheme, although evidence-based, reflects assumptions about DoD priorities that may vary across service branches or over time. Additionally, although the PASTEL framework provides a structured and transparent approach to policy evaluation, the assignment of weights and scores introduces an element of subjectivity that may influence the relative ranking of policy alternatives. Further, the analysis focused on screening and immediate prevention measures but did not fully evaluate long-term treatment and disability management policies that may be needed as screening identifies more cases. Future policy development efforts should incorporate downstream considerations, including treatment capacity, long-term care requirements, and potential impacts on medical retention standards.

An important limitation of the recommended annual PHA-integrated screening approach is the potential to miss acute Chagas disease infections. The acute phase of Chagas disease typically lasts only a few weeks to months, and annual screening intervals may not capture these transient infections. However, this limitation is mitigated by several factors: 1) acute Chagas disease is often asymptomatic, and the acute phase is time-limited (however, the infection itself persists, progressing to a chronic phase that may last a lifetime); 2) the chronic phase persists for decades and remains detectable; 3) symptomatic acute cases would likely prompt medical evaluation outside of routine screening; and 4) the primary goal of military screening is to identify chronic infections that pose long-term readiness risks. Accordingly, the proposed screening approach prioritizes detection of clinically consequential chronic disease rather than short-lived acute infections. Nonetheless, service members with suspected acute exposure (e.g., after deployment to high-risk areas or known vector contact) should receive expedited testing outside the annual PHA cycle rather than waiting for annual assessment cycles.

## CONCLUSION

Military personnel face heightened risk as a result of frequent relocations, deployments to endemic regions, and the absence of a centralized DoD policy of Chagas disease prevention, screening, and treatment. Service members could unknowingly contract Chagas disease early in their careers and later become unable to serve when chronic symptoms manifest. This disease-related attrition represents a significant loss of experience and leadership to the services. At the same time, reductions in the military health care system—such as shifting ∼155,000 beneficiaries^47^ to civilian care and closing certain facilities—underscore the need for early detection and intervention to prevent additional strain on public health resources. In this context, proactive screening and surveillance strategies represent not only a clinical priority but also a force readiness imperative.

Implementing Chagas disease testing among SMs is recommended to reduce transmission, lower mortality, and mitigate the disease burden on health care systems. This study provides a structured comparative policy analysis to inform the development of DoD-specific screening strategies tailored to the unique operational environment of the U.S. military.

This study’s decision matrix analysis provides an evidence-based framework for developing DoD policy on Chagas disease screening and prevention. The recommended Integrated PHA approach offers the optimal balance of feasibility, cost-effectiveness, and force protection benefits while building on existing military health policy infrastructure.

By leveraging existing PHA workflows, this approach enables scalable implementation without substantial disruption to clinical operations or resource allocation. Implementation should prioritize integration with current PHA protocols, development of standardized risk assessment tools, and establishment of clear testing and referral pathways for SMs identified as being at high risk. This approach ensures systematic attention to Chagas disease risk while ensuring compatibility with existing DoD health care systems to improve force health protection. Importantly, it aligns with established DoD screening paradigms, increasing the likelihood of policy adoption and sustained implementation.

Future research should evaluate the effectiveness of implemented screening protocols and assess the need for expanded treatment and long-term care policies as screening programs identify infected service members requiring medical management. Additional work is also needed to validate screening strategies using operational data and to assess potential impacts on readiness, retention, and health care utilization over time.
